# Bis(4-meth­oxy­chalcone 4-ethyl­thio­semi­carbazon­ato-κ^2^ *N* ^1^,*S*)zinc(II): crystal structure and Hirshfeld surface analysis

**DOI:** 10.1107/S2056989018000282

**Published:** 2018-01-12

**Authors:** Ming Yueh Tan, Karen A. Crouse, Thahira B. S. A. Ravoof, Mukesh M. Jotani, Edward R. T. Tiekink

**Affiliations:** a Department of Physical Sciences, Faculty of Applied Sciences and Computing, Tunku Abdul Rahman, University College, 50932 Setapak, Kuala Lumpur, Malaysia; b Department of Chemistry, Faculty of Science, Universiti Putra Malaysia, 43400, UPM Serdang, Selangor Darul Ehsan, Malaysia; c Department of Chemistry, St. Francis Xavier University, PO Box 5000, Antigonish, NS B2G 2W5, Canada; d Department of Physics, Bhavan’s Sheth R. A. College of Science, Ahmedabad, Gujarat 380001, India; e Research Centre for Crystalline Materials, School of Science and Technology, Sunway University, 47500 Bandar Sunway, Selangor Darul Ehsan, Malaysia

**Keywords:** crystal structure, zinc, hydrogen bonding, thio­semicarbazone, Hirshfeld surface analysis

## Abstract

The title Zn^II^ complex, [Zn(C_19_H_20_N_3_OS)_2_] {systematic name: bis­[(*N*-ethyl-*N*′-{(*Z*)-[(2*E*)-3-(4-meth­oxy­phen­yl)-1-phenyl­prop-2-en-1-yl­idene]amino}­carb­am­im­id­o­yl)sulfanido]zinc(II)}, features a tetra­hedrally coordinated Zn^II^ ion within an N_2_S_2_ donor set provided by two *N*,*S*-chelating thio­semicarbazone anions. The resulting five-membered Zn,C,N_2_,S chelate rings adopt different conformations, *i.e*. almost planar and an envelope with the Zn atom being the flap atom. The configuration about the imine bond within the chelate ring is *Z* but those about the exocyclic imine and ethyl­ene bonds are *E*. In the crystal, supra­molecular [100] chains mediated by thio­amide-N—H⋯S(thione) hydrogen bonds and eight-membered thio­amide {⋯HNCS}_2_ synthons are observed. A range of inter­actions, including C—H⋯O, C—H⋯π, C—H⋯π(chelate ring) and π(meth­oxy­benzene)—π(chelate ring) consolidate the packing. The Hirshfeld surface analysis performed on the title complex also indicates the influence of the inter­actions involving the chelate rings upon the packing along with the more conventional contacts.

## Abstract 2

The title thio­semicarbazone compound features tetra­hedrally coordinated Zn^II^ atoms within N_2_S_2_ donor sets because of the presence of chelating thio­semicarbazone anions. Supra­molecular chains are found in the crystal owing to the formation of thio­amide-N—H⋯S(thione) hydrogen bonds and eight-membered thio­amide {⋯HNCS}_2_ synthons.

## Chemical context   

With potentially five different substituents, thio­semicarbazone derivatives, *R* ^1^ *R* ^2^C=N—N(*R* ^3^)—C(=S)N*R* ^4^ *R* ^5^ for *R* ^1–5^ = H/alk­yl/aryl, are numerous and multi-functional. Their preparation is often facile, being formed from the condensation reaction between an aldehyde (or a ketone) with the amine group of a thio­semicarbazide precursor. In the same way, the diversity in ligand construction ensures a rich coord­ination chemistry (Lobana *et al.*, 2009 ▸). A primary motivation for investigating metal complexes of thio­semi­carbazones and related derivatives rests with their putative biological activity (Espíndola *et al.*, 2015 ▸; Pelivan, *et al.*, 2016 ▸; Low *et al.*, 2016 ▸; Bisceglie *et al.*, 2018 ▸). Thus, promising activity has been exhibited by various metal complexes against a range of diseases (Dilworth & Hueting, 2012 ▸). In the context of the present report, it is noteworthy that Zn^II^ thio­semicarbazone complexes have been explored as therapeutics for the treatment of cancer (Afrasiabi *et al.*, 2003 ▸), viral diseases (Garoufis *et al.*, 2009 ▸) and bacterial infections (Quiroga & Ranninger, 2004 ▸). Such considerations motivate our inter­est in this class of compound (Yusof *et al.*, 2015 ▸). Herein, in contin­uation of our structural studies of Zn^II^ thio­semi­carbazones (Tan *et al.*, 2017 ▸), the X-ray crystal structure of the title compound, (I), is described along with an analysis of its Hirshfeld surfaces in order to gain more information on the mode of association between mol­ecules in the mol­ecular packing.
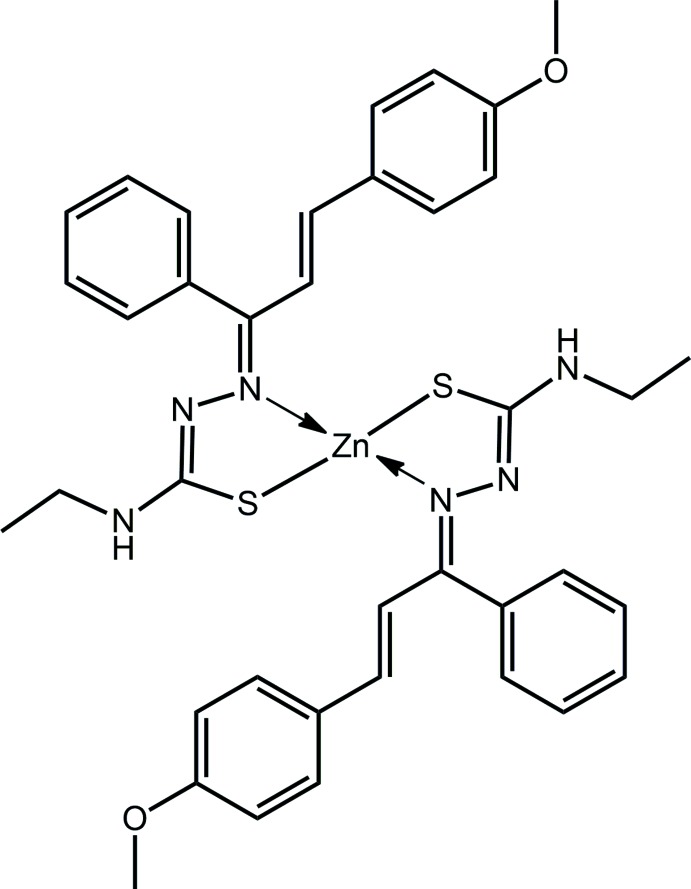


## Structural commentary   

**Figure 1:**
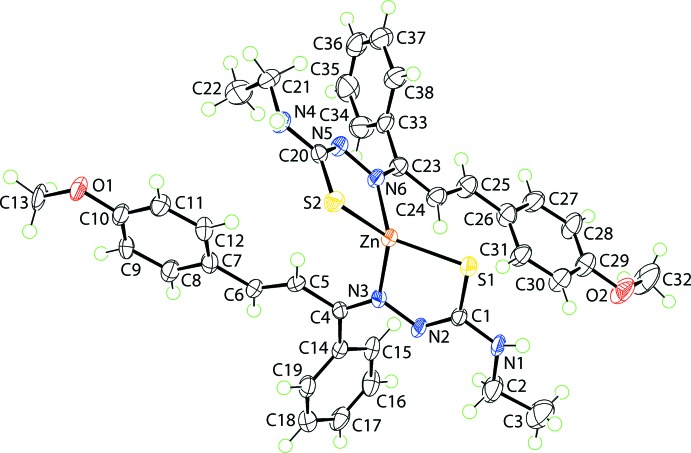
The mol­ecular structure of (I) showing the atom-labelling scheme and displacement ellipsoids at the 70% probability level.

The mol­ecular structure of (I), Fig. 1 ▸, sees the Zn^II^ atom coordinated by two chelating thio­semicarbazone anions, each *via* the thiol­ate-S and imine-N atoms, Table 1 ▸. The resulting N_2_S_2_ donor set defines a distorted tetra­hedral geometry, with the range of angles subtended at the zinc atom being an acute 87.29 (9)° for the S1—Zn—N3 chelate angle to 127.92 (4)° for S1—Zn—S2. The assignment of four-coordinate geometries can be qu­anti­fied by comparing the calculated value of τ_4_, in this case 0.74, with the ideal values for an ideal tetra­hedron, *i.e*. 1.00, and perfect square-planar geometry, *i.e*. 0.00 (Yang *et al.*, 2007 ▸), indicating a distorted tetra­hedral geometry in (I). The configuration about each of the endocyclic imine bonds is *Z*, because of the dictates of chelation. By contrast, each of the exocyclic imine C=N bonds is *E*, as are the configurations about the ethyl­ene bonds, Table 1 ▸.

The mode of the coordination of the thio­semicarbazone ligands leads to the formation of five-membered ZnSCN_2_ chelate rings, and these adopt different conformations. Whereas, the (Zn,S1,C1,N2,N3) ring is almost planar (r.m.s. deviation = 0.0325 Å), the (Zn,S2,C20,N5,N6) chelate ring is best described as an envelope with the Zn atom lying 0.205 (5) Å out of the plane of the remaining four atoms (r.m.s. deviation = 0.0011 Å). The dihedral angle between the mean planes through the chelate rings is 79.68 (8)°. To a first approximation, the thio­semicarbazone ligands comprise two planar regions. Thus, the non-hydrogen, non-phenyl atoms of the atoms of the S1-ligand define one plane (r.m.s. deviation = 0.1910 Å), which forms a dihedral angle of 54.53 (8)° with the (C14–C19) ring, consistent with a near perpendicular relationship. The comparable values for the S2-ligand are 0.2800 Å and 75.09 (11)°, respectively.

## Supra­molecular features   

**Figure 2:**
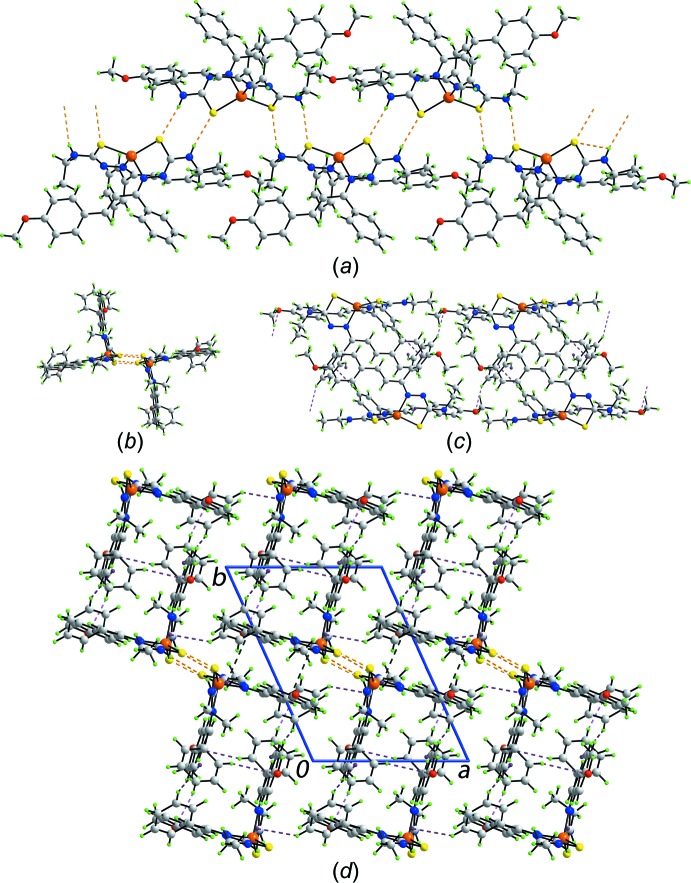
Mol­ecular packing in (I): (*a*) a view of the linear supra­molecular chain sustained by thio­amide-N—H⋯S(thiol­ate) hydrogen bonds shown as orange dashed lines, (*b*) a view of the supra­molecular chain down the axis of propagation, (*c*) a side-on view of the centrosymmetric supra­molecular tube stabilized by C—H⋯O (pink dashed lines) and C—H⋯π (purple dashed lines) inter­actions and (*d*) a view of the unit-cell contents shown in projection down the *c* axis showing C—H⋯(chelate ring) and π(chelate ring)–π(arene) inter­actions as as purple and black dashed lines, respectively.

The most prominent feature of the mol­ecular packing is the formation of supra­molecular chains along the *c-*axis direction sustained by eight-membered thio­amide {⋯HNCS}_2_ synthons, Fig. 2 ▸ *a* and Table 2 ▸. When the array is viewed down the axis of propagation, Fig. 2 ▸ *b*, it is evident that two rows of mol­ecules, each with a right-angle topology, are connected by N—H⋯S(thione) hydrogen bonds. Centrosymmetrically related right angles are connected into a supra­molecular tube, Fig. 2 ▸ *c*, *via* imine-phenyl-C—H⋯O(meth­oxy), imine-phenyl-C—H⋯π(imine-phen­yl) and imine-phenyl-C—H⋯π(meth­oxy­benzene) inter­actions, Table 2 ▸. The connections between the tubes over and above the hydrogen bonding involve chelate rings, which are more and more being recognized as being important in consolidating crystal structures (Tiekink, 2017 ▸). The first kind of inter­action is of the type imine-phenyl-C—H⋯(chelate ring) where the chelate ring is defined by the five-membered (Zn,S2,C20,N5,N6) grouping which, as mentioned above, is non-planar, indicating that aromaticity is not the sole criterion for the formation of C—H⋯(chelate ring) inter­actions (Palusiak & Krygowski, 2007 ▸; Yeo *et al.*, 2014 ▸; Zukerman-Schpector *et al.*, 2016 ▸). The second contact between tubes involving chelate rings is of the type π(Zn,S1,C1,N2,N3)–π(C7–C12)^v^ with a ring centroid–ring centroid separation of 3.778 (2) Å and angle of inclination = 15.04 (17)° for symmetry operation (v): 2 − *x*, 1 − *y*, 1 − *z*. A review has appeared very recently on the topic of π(chelate ring)–π(arene) and π(chelate ring)–π(chelate ring) inter­actions where it was suggested that inter­actions of the former type provide comparable energies of stabilization to mol­ecular packing as do weak conventional hydrogen bonds (Malenov *et al.*, 2017 ▸). A view of the unit-cell contents is shown in Fig. 2 ▸ *d*.

## Analysis of the Hirshfeld surfaces   

**Figure 3:**
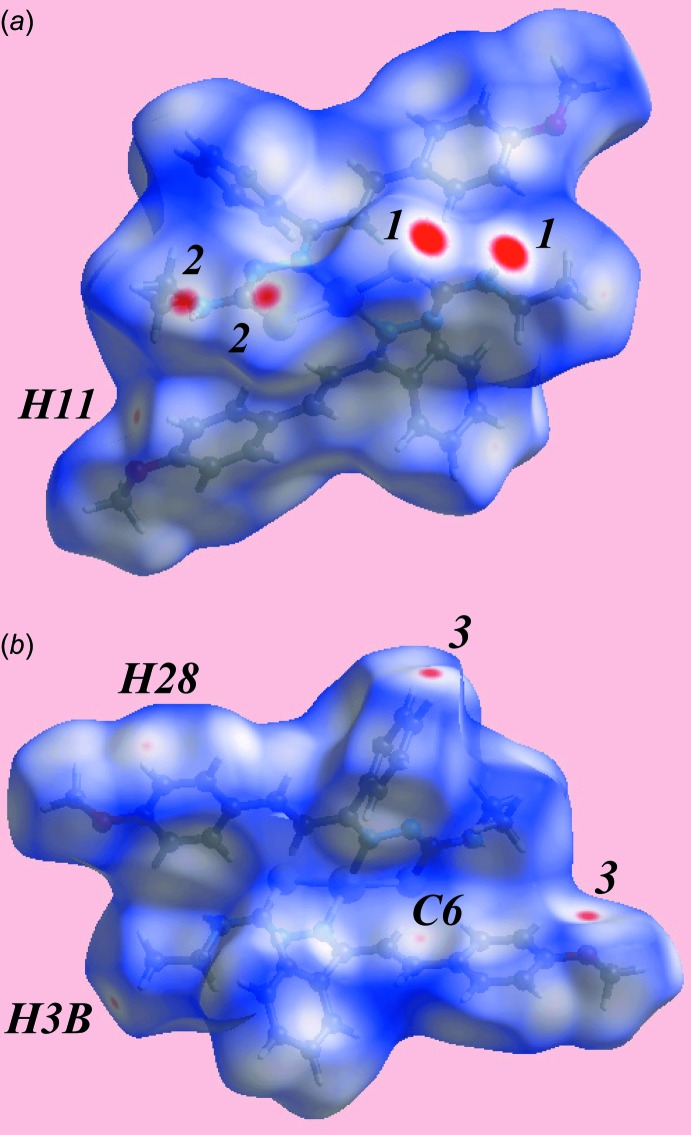
Two views of Hirshfeld surface mapped over *d*_norm_ for (I) in the range −0.152 to +1.534 au.

**Figure 4:**
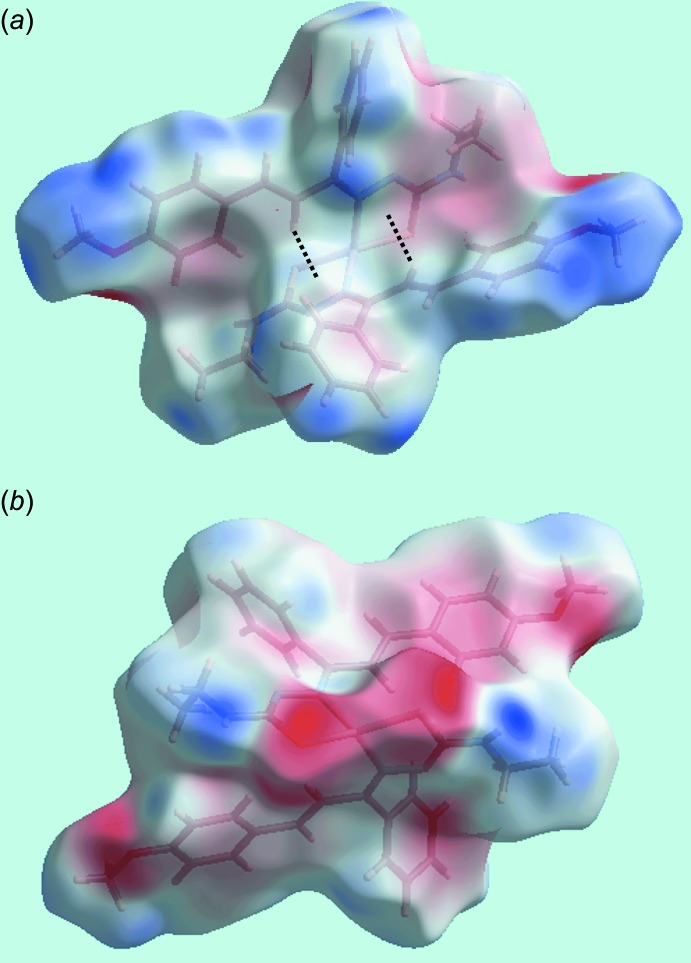
Two views of Hirshfeld surface mapped over the electrostatic potential for (I) in the range ± 0.051 au highlighting intra­molecular C—H⋯π(chelate) inter­actions as black dotted lines.

**Figure 5:**
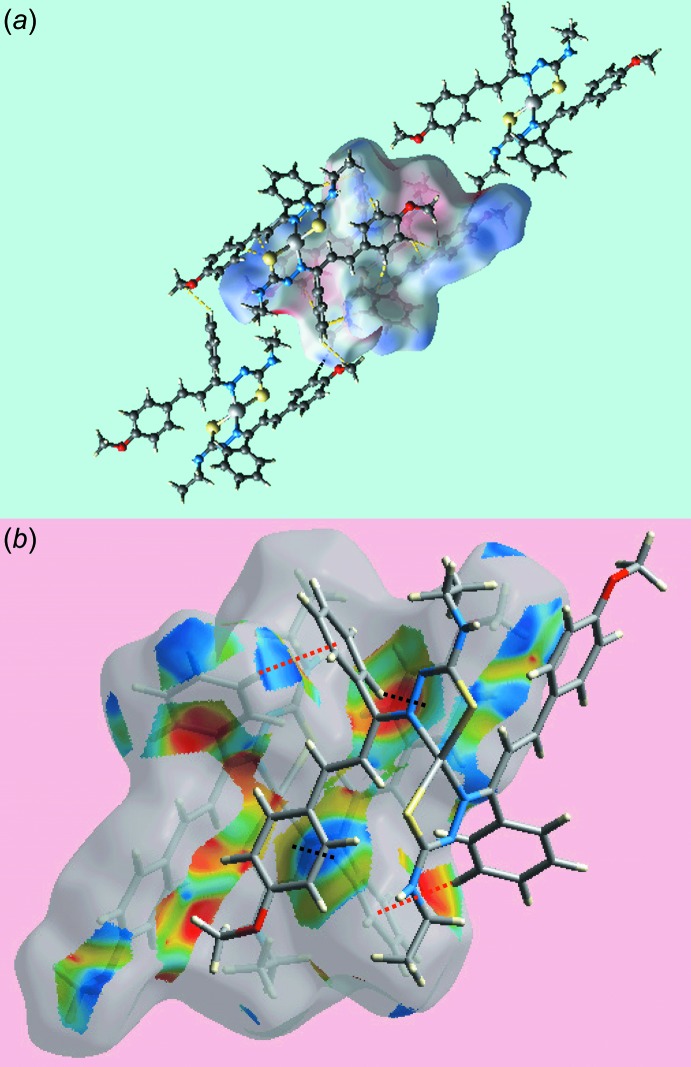
Views of Hirshfeld surface about reference mol­ecule of (I) mapped (*a*) over the electrostatic potential highlighting short inter­atomic H⋯H and C⋯H/H⋯C contacts by red and yellow dashed lines, respectively, and (*b*) with the shape-index property highlighting C—H⋯π/π⋯H—C contacts involving imine-phenyl and meth­oxy-benzene rings by red and black dashed lines, respectively.

The Hirshfeld surfaces calculated for (I) were performed in accord with recent work on a related complex (Tan *et al.*, 2017 ▸) and provide more insight into the inter­molecular inter­actions occurring in the crystal. The donors and acceptors of the inter­molecular N—H⋯S hydrogen bonds are viewed as bright-red spots, labelled as ‘1’ and ‘2’ in Fig. 3 ▸ *a*, and the inter­molecular C—H⋯O contacts appear as tiny red spots with label ‘3’ in Fig. 3 ▸ *b* on the Hirshfeld surface mapped over *d*_norm_. The faint-red spots near the H3*B*, H11, H28 and C6 sites represent significant short inter­atomic H⋯H and C⋯H/H⋯C contacts, Fig. 3 ▸ and Table 3 ▸. The structure features two intra­molecular C—H⋯π(chelate) contacts, *i.e*. between ethyl­ene-C5—H and the (Zn,S2,C20,N5,N6) ring and between ethyl­ene-C24—H and the (Zn,S1,C1,N2,N3) ring, Table 2 ▸, which are viewed as blue and red regions assigned to positive and negative potentials, respectively, on the Hirshfeld surfaces mapped over electrostatic potential and are highlighted in Fig. 4 ▸ *a*. The donors and acceptors of the inter­molecular N—H⋯S and C—H⋯O inter­actions are also viewed as blue and red regions about respective atoms in the images of Fig. 4 ▸. The C—H⋯π inter­actions involving imine-phenyl and meth­oxy-benzene rings are evident in short inter­atomic C⋯H/H⋯C contacts, Table 3 ▸. The views of Hirshfeld surfaces about a reference mol­ecule mapped over the electrostatic potential highlighting short inter­atomic H⋯H and C⋯H/H⋯C contacts and that mapped within the shape-index property highlighting C—H⋯π/π⋯H—C contacts are illustrated in Fig. 5 ▸ *a* and *b*, respectively.

**Figure 6:**
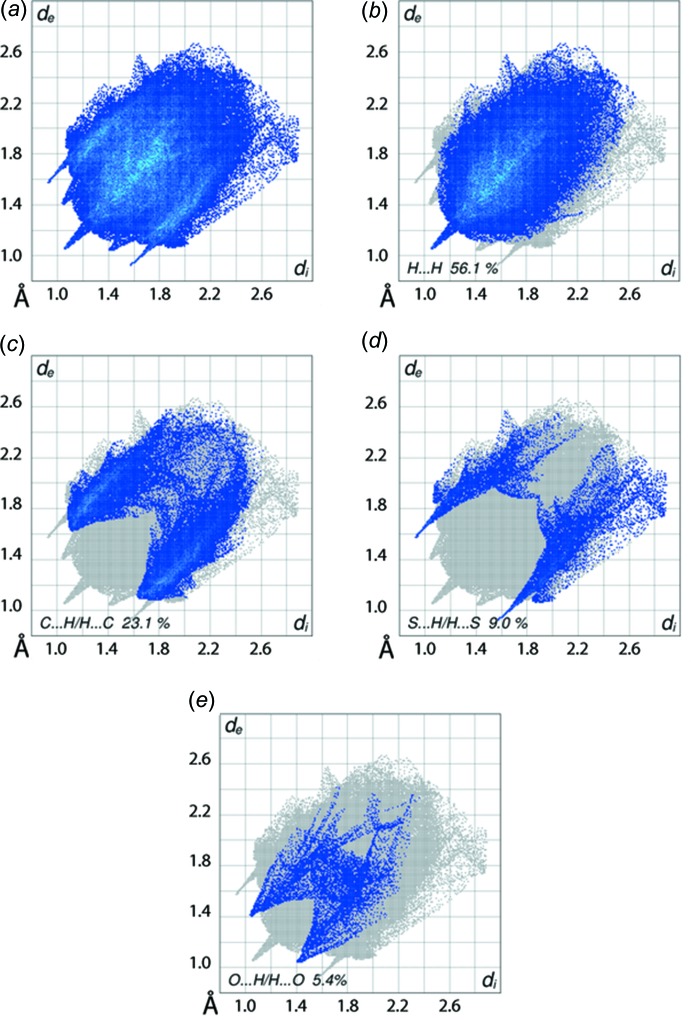
(*a*) The full two-dimensional fingerprint plot and fingerprint plots delineated into (*b*) H⋯H, (*c*) C⋯H/H⋯C, (*d*) S⋯H/H⋯S and (*e*) O⋯H/H⋯O contacts for (I).

The overall two dimensional fingerprint plot for (I), Fig. 6 ▸ *a*, and those delineated into H⋯H, C⋯H/H⋯C, S⋯H/H⋯S and O⋯H/H⋯O contacts (McKinnon *et al.*, 2007 ▸) are shown in Fig. 6 ▸ *b*–*e* and illustrate the influence of various inter­molecular inter­actions instrumental in the crystal of (I). The percentage contributions from the different inter­atomic contacts to the Hirshfeld surface are summarized in Table 4 ▸. The single spike in the centre at *d*_e_ + *d*_i_ ∼ 2.1 Å in Fig. 6 ▸ *a* is due to a short inter­atomic H⋯H contact (Table 3 ▸) and the two pairs of spikes about this central spike, at *d*_e_ + *d*_i_ ∼ 2.6 Å, indicate the inter­molecular C—H⋯O and N—H⋯S inter­actions, Fig. 6 ▸ *c*,*d*. The points related to short inter­atomic O⋯H/H⋯O contacts listed in Table 3 ▸ are merged within the respective plot of Fig. 6 ▸ *e*. The C⋯H/H⋯C contacts provide the second greatest contribution to the Hirshfeld surface, Table 4 ▸. This is due to the combined effect of short inter­atomic C⋯H/H⋯C contacts (Table 3 ▸) in addition to C—H⋯π contacts, summarized in Table 2 ▸. The most significant short atomic C6⋯H28 contact is evident from a pair of short peaks at *d*_e_ + *d*_i_ ∼ 2.7 Å in the fingerprint plot delineated into C⋯H/H⋯C contacts, Fig. 6 ▸ *c*. The short inter­atomic contact between the Zn^II^ atom and imine-phenyl-C18 and H18 atoms, Table 3 ▸, and the contribution of 0.6% from Zn⋯H/H⋯Zn and Zn⋯C/C⋯Zn contacts to the Hirshfeld surface, Table 4 ▸, reflect the presence of inter­molecular C—H⋯π(chelate) inter­actions in the crystal. The π(chelate)–π(benzene) contacts described in the *Supra­molecular features* section (§3) are also reflected from the small but important contribution from C⋯N/N⋯C and C⋯S/S⋯C contacts, Table 4 ▸, to the Hirshfeld surface of (I).

## Database survey   

The most relevant structure available for comparison is that of the recently described bis­(*N*′-{(*E*)-[(2*E*)-1,3-di­phenyl­prop-2-en-1-yl­idene]-amino}-*N*-ethyl­carbamimido­thio­ato-κ^2^ *N*′,*S*)zinc(II) mol­ecule, which differs from (I) in that there are no additional substituents in the phenyl ring appended at the ethyl­ene bond (Tan *et al.*, 2017 ▸). Similar tetra­hedral N_2_S_2_ coordination geometries are found with values of τ_4_ of 0.70 and 0.74 for the two independent mol­ecules comprising the asymmetric unit. Indeed, in the publication describing this structure (Tan *et al.*, 2017 ▸), it was mentioned there are nine structures in the literature conforming to the general formula Zn[SC(NH*R*)=NN=C*R*′*R*′′]_2_ and all structures adopt the same basic structural motif as described herein for (I).

## Synthesis and crystallization   

Analytical grade reagents were used as procured and without further purification. 4-Ethyl-3-thio­semicarbazide (1.1919 g, 0.01 mol) and 4-meth­oxy­chalcone (2.3828 g, 0.01 mol) were dissolved separately in hot absolute ethanol (30 ml) and mixed while stirring. About five drops of concentrated hydro­chloric acid were added to the mixture to catalyse the reaction. The reaction mixture was heated and stirred for about 20 min, and stirring was continued for another 30 min at room temperature. The resulting yellow precipitate, 4-meth­oxy­chalcone-4-ethyl-3-thio­semicarbazone, was filtered off, washed with cold absolute ethanol and dried *in vacuo* after which it was used without further purification. 4-Meth­oxy­chalcone-4-ethyl-3-thio­semicarbazone (0.3395 g, 0.01 mol) was dissolved in hot absolute ethanol (30 ml), which was added to a solution of Zn(CH_3_COO)_2_·2H_2_O (0.1098 g, 0.50 mmol) in hot absolute ethanol (40 ml). The mixture was heated and stirred for about 10 min, followed by stirring for 1 h at room temperature. The yellow precipitate obtained was filtered, washed with cold ethanol and dried *in vacuo*. Single crystals were grown at room temperature from the slow evaporation of the title compound in a mixed solvent system containing di­methyl­formamide and aceto­nitrile (1:1; *v*/*v* 20 ml). IR (cm^−1^): 3351 ν(N—H), 1597 ν(C=N), 1009 ν(N—N), 420 ν(*M*—N), 362 ν(*M*—S).

## Refinement   

Crystal data, data collection and structure refinement details are summarized in Table 5 ▸. The carbon-bound H atoms were placed in calculated positions (C—H = 0.95–0.99 Å) and were included in the refinement in the riding-model approximation, with *U*_iso_(H) set to 1.2–1.5*U*_eq_(C). The nitro­gen-bound H atoms were located in a difference-Fourier map but were refined with a distance restraint of N—H = 0.88±0.01 Å, and with *U*_iso_(H) set to 1.2*U*_eq_(N). The maximum and minimum residual electron density peaks of 1.10 and 0.59 e Å^−3^, respectively, are located 1.04 and 0.71 Å from the Zn atom.

## Supplementary Material

Crystal structure: contains datablock(s) I, global. DOI: 10.1107/S2056989018000282/hb7725sup1.cif

Structure factors: contains datablock(s) I. DOI: 10.1107/S2056989018000282/hb7725Isup2.hkl

CCDC reference: 1814817

Additional supporting information:  crystallographic information; 3D view; checkCIF report

## Figures and Tables

**Table 1:** Selected bond lengths (Å)

Zn—N3	2.041 (3)	C4—N3	1.310 (5)
Zn—N6	2.071 (3)	C5—C6	1.349 (5)
Zn—S1	2.2879 (11)	C20—N5	1.307 (5)
Zn—S2	2.2757 (11)	C23—N6	1.319 (5)
C1—N2	1.314 (5)	C24—C25	1.344 (5)

**Table 2:** Hydrogen-bond geometry (Å, °) *Cg*1—*Cg*4 are the centroids of the (C33–C38), (Zn,S2,C20,N5,N6), (C26—C31) and (Zn,S1,C1,N2,N3) rings, respectively.

*D*—H⋯*A*	*D*—H	H⋯*A*	*D*⋯*A*	*D*—H⋯*A*
N1—H1*N*⋯S1^i^	0.85 (5)	2.66 (5)	3.506 (4)	171 (3)
N4—H4*N*⋯S2^ii^	0.84 (5)	2.82 (5)	3.477 (5)	137 (4)
C36—H36⋯O1^iii^	0.95	2.57	3.428 (6)	151
C16—H16⋯*Cg*1^iv^	0.95	2.85	3.747 (4)	157
C18—H18⋯*Cg*2^v^	0.95	2.69	3.485 (5)	141
C34—H34⋯*Cg*3^iv^	0.95	2.72	3.555 (6)	148
C5—H5⋯*Cg*2	0.95	2.67	3.462 (5)	142
C24—H24⋯*Cg*4	0.95	2.55	3.421 (5)	153

Symmetry codes: (i) 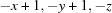; (ii) 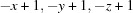; (iii) 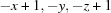; (iv) 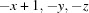; (v) 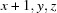.

**Table 3:** Summary of short inter-atomic contacts (Å) in (I)

Contact	Distance	Symmetry operation
H3*B*⋯H11	2.11	*x*, *y*, − 1 + *z*
Zn⋯H18	2.93	− 1 + *x*, *y*, *z*
Zn⋯C18	3.871 (8)	− 1 + *x*, *y*, *z*
O2⋯H22*B*	2.56	*x*, *y*, − 1 + *z*
C6⋯H28	2.74	1 − *x*, − *y*, − *z*
C7⋯H28	2.85	1 − *x*, − *y*, − *z*
C15⋯H27	2.80	1 − *x*, − *y*, − *z*
C17⋯H38	2.79	1 + *x*, *y*, *z*
C24⋯H17	2.78	− 1 + *x*, *y*, *z*
C26⋯H34	2.81	1 − *x*, − *y*, − *z*
C30⋯H35	2.84	1 − *x*, − *y*, − *z*
C31⋯H34	2.80	1 − *x*, − *y*, − *z*
C36⋯H16	2.85	1 − *x*, − *y*, − *z*
C37⋯H16	2.83	1 − *x*, − *y*, − *z*

**Table 4:** Percentage contributions of inter-atomic contacts to the Hirshfeld surface for (I)

Contact	Percentage contribution
H⋯H	56.1
C⋯H/H⋯C	23.1
S⋯H/H⋯S	9.0
O⋯H/H⋯O	5.4
N⋯H/H⋯N	1.6
C⋯S/S⋯C	1.3
C⋯N/N⋯C	1.1
Zn⋯H/H⋯Zn	0.6
Zn⋯C/C⋯Zn	0.6
C⋯C	0.6
C⋯O/O⋯C	0.3
N⋯O/O⋯N	0.3

**Table 5:** Experimental details

Crystal data	
Chemical formula	[Zn(C_19_H_20_N_3_OS)_2_]
*M*_r_	742.25
Crystal system, space group	Triclinic, *P* 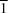
Temperature (K)	100
*a*, *b*, *c* (Å)	10.5013 (6), 14.2836 (8), 14.8282 (9)
α, β, γ (°)	107.173 (5), 108.152 (5), 106.259 (5)
*V* (Å^3^)	1842.0 (2)
*Z*	2
Radiation type	Mo *K*α
μ (mm^−1^)	0.82
Crystal size (mm)	0.25 × 0.15 × 0.05

Data collection	
Diffractometer	Agilent Technologies SuperNova Dual diffractometer with Atlas detector
Absorption correction	Multi-scan (*CrysAlis PRO*; Agilent, 2013 ▸)
*T*_min_, *T*_max_	0.887, 1.000
No. of measured, independent and observed [*I* > 2σ(*I*)] reflections	19299, 8464, 5619
*R*_int_	0.071
(sin θ/λ)_max_ (Å^−1^)	0.650

Refinement	
*R*[*F* ^2^ > 2σ(*F* ^2^)], *wR*(*F* ^2^), *S*	0.065, 0.171, 1.01
No. of reflections	8464
No. of parameters	452
H-atom treatment	H atoms treated by a mixture of independent and constrained refinement
Δρ_max_, Δρ_min_ (e Å^−3^)	1.10, −0.59

Computer programs: *CrysAlis PRO* (Agilent, 2013 ▸), *SHELXS97* (Sheldrick, 2008 ▸), *SHELXL2014* (Sheldrick, 2015 ▸), *ORTEP-3 for Windows* (Farrugia, 2012 ▸), *DIAMOND* (Brandenburg, 2006 ▸) and *publCIF* (Westrip, 2010 ▸).

## supplementary crystallographic information

### Crystal data

**Table:**

[Zn(C_19_H_20_N_3_OS)_2_]	*Z* = 2
*M**_r_* = 742.25	*F*(000) = 776
Triclinic, *P*1	*D*_x_ = 1.338 Mg m^−^^3^
*a* = 10.5013 (6) Å	Mo *K*α radiation, λ = 0.71073 Å
*b* = 14.2836 (8) Å	Cell parameters from 4004 reflections
*c* = 14.8282 (9) Å	θ = 2.8–27.5°
α = 107.173 (5)°	µ = 0.82 mm^−^^1^
β = 108.152 (5)°	*T* = 100 K
γ = 106.259 (5)°	Prism, yellow
*V* = 1842.0 (2) Å^3^	0.25 × 0.15 × 0.05 mm

### Data collection

**Table:**

Agilent Technologies SuperNova Dual diffractometer with Atlas detector	8464 independent reflections
Radiation source: SuperNova (Mo) X-ray Source	5619 reflections with *I* > 2σ(*I*)
Mirror monochromator	*R*_int_ = 0.071
Detector resolution: 10.4041 pixels mm^-1^	θ_max_ = 27.5°, θ_min_ = 2.9°
ω scan	*h* = −13→13
Absorption correction: multi-scan (CrysAlis PRO; Agilent, 2013)	*k* = −18→18
*T*_min_ = 0.887, *T*_max_ = 1.000	*l* = −19→19
19299 measured reflections	

### Refinement

**Table:**

Refinement on *F*^2^	0 restraints
Least-squares matrix: full	H atoms treated by a mixture of independent and constrained refinement
*R*[*F*^2^ > 2σ(*F*^2^)] = 0.065	*w* = 1/[σ^2^(*F*_o_^2^) + (0.066*P*)^2^ + 1.1328*P*] where *P* = (*F*_o_^2^ + 2*F*_c_^2^)/3
*wR*(*F*^2^) = 0.171	(Δ/σ)_max_ = 0.001
*S* = 1.01	Δρ_max_ = 1.10 e Å^−^^3^
8464 reflections	Δρ_min_ = −0.59 e Å^−^^3^
452 parameters	

### Special details

**Table:**

Geometry. All esds (except the esd in the dihedral angle between two l.s. planes) are estimated using the full covariance matrix. The cell esds are taken into account individually in the estimation of esds in distances, angles and torsion angles; correlations between esds in cell parameters are only used when they are defined by crystal symmetry. An approximate (isotropic) treatment of cell esds is used for estimating esds involving l.s. planes.
Refinement. The maximum and minimum residual electron density peaks of 1.10 and 0.59 eÅ^-3^, respectively, were located 1.04 Å and 0.71 Å from the Zn atom.

### Fractional atomic coordinates and isotropic or equivalent isotropic displacement parameters (Å^2^)

**Table:**

	*x*	*y*	*z*	*U*_iso_*/*U*_eq_	
Zn	0.61283 (5)	0.39864 (3)	0.26017 (3)	0.01731 (14)	
S1	0.53424 (11)	0.44366 (8)	0.12335 (8)	0.0199 (2)	
S2	0.63421 (11)	0.47815 (8)	0.42482 (7)	0.0209 (2)	
O1	1.0784 (3)	0.3417 (2)	0.8198 (2)	0.0285 (7)	
O2	0.2660 (4)	0.0652 (2)	−0.4057 (2)	0.0383 (8)	
N1	0.6593 (4)	0.4265 (3)	−0.0084 (2)	0.0211 (8)	
H1N	0.615 (5)	0.463 (3)	−0.029 (3)	0.025*	
N2	0.7664 (4)	0.3874 (2)	0.1248 (2)	0.0192 (7)	
N3	0.7733 (3)	0.3790 (2)	0.2167 (2)	0.0162 (7)	
N4	0.5490 (4)	0.3597 (3)	0.5203 (2)	0.0221 (8)	
H4N	0.556 (5)	0.420 (3)	0.556 (3)	0.027*	
N5	0.4901 (4)	0.2575 (3)	0.3512 (2)	0.0201 (7)	
N6	0.4944 (4)	0.2545 (2)	0.2579 (2)	0.0186 (7)	
C1	0.6656 (4)	0.4179 (3)	0.0811 (3)	0.0163 (8)	
C2	0.7722 (5)	0.4190 (4)	−0.0435 (3)	0.0346 (11)	
H2A	0.7767	0.3485	−0.0534	0.042*	
H2B	0.8689	0.4758	0.0106	0.042*	
C3	0.7415 (7)	0.4310 (5)	−0.1443 (4)	0.0499 (14)	
H3A	0.6472	0.3735	−0.1986	0.075*	
H3B	0.8198	0.4266	−0.1657	0.075*	
H3C	0.7373	0.5008	−0.1347	0.075*	
C4	0.8760 (4)	0.3507 (3)	0.2610 (3)	0.0160 (8)	
C5	0.8940 (4)	0.3512 (3)	0.3620 (3)	0.0188 (8)	
H5	0.8345	0.3757	0.3913	0.023*	
C6	0.9879 (4)	0.3199 (3)	0.4181 (3)	0.0183 (8)	
H6	1.0445	0.2925	0.3877	0.022*	
C7	1.0097 (4)	0.3247 (3)	0.5222 (3)	0.0203 (9)	
C8	1.1094 (5)	0.2881 (3)	0.5709 (3)	0.0246 (9)	
H8	1.1610	0.2602	0.5355	0.030*	
C9	1.1352 (4)	0.2915 (3)	0.6701 (3)	0.0241 (9)	
H9	1.2025	0.2653	0.7015	0.029*	
C10	1.0617 (5)	0.3334 (3)	0.7223 (3)	0.0233 (9)	
C11	0.9624 (5)	0.3715 (3)	0.6745 (3)	0.0238 (9)	
H11	0.9126	0.4012	0.7105	0.029*	
C12	0.9368 (4)	0.3663 (3)	0.5766 (3)	0.0223 (9)	
H12	0.8681	0.3915	0.5450	0.027*	
C13	1.1781 (5)	0.3025 (3)	0.8702 (3)	0.0309 (11)	
H13A	1.1450	0.2258	0.8276	0.046*	
H13B	1.1807	0.3128	0.9392	0.046*	
H13C	1.2766	0.3420	0.8782	0.046*	
C14	0.9722 (4)	0.3227 (3)	0.2124 (3)	0.0186 (8)	
C15	0.9112 (5)	0.2427 (3)	0.1112 (3)	0.0228 (9)	
H15	0.8075	0.2064	0.0726	0.027*	
C16	1.0016 (5)	0.2162 (3)	0.0672 (3)	0.0266 (10)	
H16	0.9595	0.1606	−0.0011	0.032*	
C17	1.1524 (5)	0.2698 (3)	0.1217 (3)	0.0275 (10)	
H17	1.2137	0.2525	0.0902	0.033*	
C18	1.2141 (5)	0.3488 (3)	0.2223 (3)	0.0280 (10)	
H18	1.3179	0.3849	0.2603	0.034*	
C19	1.1250 (4)	0.3755 (3)	0.2677 (3)	0.0224 (9)	
H19	1.1679	0.4299	0.3368	0.027*	
C20	0.5495 (4)	0.3535 (3)	0.4270 (3)	0.0184 (8)	
C21	0.4684 (5)	0.2645 (3)	0.5309 (3)	0.0299 (10)	
H21A	0.3728	0.2218	0.4686	0.036*	
H21B	0.4480	0.2881	0.5928	0.036*	
C22	0.5510 (6)	0.1933 (4)	0.5426 (4)	0.0411 (12)	
H22A	0.5634	0.1641	0.4788	0.062*	
H22B	0.4950	0.1342	0.5543	0.062*	
H22C	0.6477	0.2359	0.6023	0.062*	
C23	0.4179 (4)	0.1593 (3)	0.1782 (3)	0.0200 (9)	
C24	0.4155 (4)	0.1516 (3)	0.0785 (3)	0.0216 (9)	
H24	0.4833	0.2114	0.0780	0.026*	
C25	0.3253 (5)	0.0669 (3)	−0.0139 (3)	0.0248 (9)	
H25	0.2647	0.0039	−0.0132	0.030*	
C26	0.3140 (5)	0.0651 (3)	−0.1153 (3)	0.0249 (10)	
C27	0.2184 (5)	−0.0286 (3)	−0.2070 (3)	0.0295 (10)	
H27	0.1651	−0.0911	−0.2018	0.035*	
C28	0.1998 (5)	−0.0322 (3)	−0.3052 (3)	0.0318 (11)	
H28	0.1346	−0.0966	−0.3661	0.038*	
C29	0.2763 (5)	0.0582 (3)	−0.3139 (3)	0.0299 (10)	
C30	0.3721 (5)	0.1522 (3)	−0.2241 (3)	0.0282 (10)	
H30	0.4247	0.2148	−0.2295	0.034*	
C31	0.3900 (5)	0.1540 (3)	−0.1278 (3)	0.0259 (10)	
H31	0.4567	0.2184	−0.0673	0.031*	
C32	0.1792 (7)	−0.0321 (4)	−0.5000 (3)	0.0525 (16)	
H32A	0.2138	−0.0877	−0.4931	0.079*	
H32B	0.1885	−0.0185	−0.5591	0.079*	
H32C	0.0759	−0.0563	−0.5120	0.079*	
C33	0.3334 (5)	0.0649 (3)	0.1917 (3)	0.0218 (9)	
C34	0.3828 (5)	−0.0154 (3)	0.1916 (4)	0.0319 (11)	
H34	0.4668	−0.0129	0.1790	0.038*	
C35	0.3087 (5)	−0.0999 (4)	0.2103 (4)	0.0385 (12)	
H35	0.3436	−0.1544	0.2112	0.046*	
C36	0.1864 (5)	−0.1056 (3)	0.2271 (3)	0.0327 (11)	
H36	0.1371	−0.1633	0.2401	0.039*	
C37	0.1351 (5)	−0.0266 (3)	0.2251 (3)	0.0317 (11)	
H37	0.0494	−0.0306	0.2357	0.038*	
C38	0.2088 (5)	0.0584 (3)	0.2076 (3)	0.0274 (10)	
H38	0.1733	0.1125	0.2065	0.033*	

### Atomic displacement parameters (Å^2^)

**Table:**

	*U*^11^	*U*^22^	*U*^33^	*U*^12^	*U*^13^	*U*^23^
Zn	0.0161 (2)	0.0230 (2)	0.0138 (2)	0.00749 (18)	0.00647 (18)	0.00934 (18)
S1	0.0182 (5)	0.0282 (5)	0.0191 (5)	0.0120 (4)	0.0084 (4)	0.0146 (4)
S2	0.0217 (5)	0.0230 (5)	0.0160 (5)	0.0072 (4)	0.0089 (4)	0.0068 (4)
O1	0.0313 (17)	0.0351 (16)	0.0156 (14)	0.0077 (13)	0.0071 (13)	0.0147 (13)
O2	0.050 (2)	0.0385 (17)	0.0188 (16)	0.0078 (16)	0.0142 (15)	0.0127 (14)
N1	0.0229 (19)	0.0325 (19)	0.0167 (17)	0.0154 (15)	0.0089 (14)	0.0173 (15)
N2	0.0205 (18)	0.0293 (17)	0.0120 (16)	0.0113 (14)	0.0083 (13)	0.0117 (14)
N3	0.0156 (16)	0.0206 (16)	0.0112 (15)	0.0069 (13)	0.0046 (13)	0.0068 (13)
N4	0.0245 (19)	0.0297 (18)	0.0118 (16)	0.0102 (16)	0.0098 (14)	0.0070 (14)
N5	0.0214 (18)	0.0260 (17)	0.0140 (16)	0.0093 (14)	0.0083 (14)	0.0095 (14)
N6	0.0195 (17)	0.0263 (17)	0.0159 (16)	0.0117 (14)	0.0096 (13)	0.0121 (14)
C1	0.018 (2)	0.0170 (18)	0.0117 (18)	0.0046 (15)	0.0047 (15)	0.0066 (15)
C2	0.039 (3)	0.054 (3)	0.024 (2)	0.027 (2)	0.018 (2)	0.024 (2)
C3	0.055 (4)	0.076 (4)	0.040 (3)	0.032 (3)	0.032 (3)	0.035 (3)
C4	0.0141 (19)	0.0165 (17)	0.0130 (18)	0.0033 (15)	0.0031 (15)	0.0064 (15)
C5	0.017 (2)	0.027 (2)	0.0177 (19)	0.0095 (16)	0.0106 (16)	0.0125 (16)
C6	0.020 (2)	0.0224 (19)	0.0132 (18)	0.0077 (16)	0.0076 (15)	0.0081 (16)
C7	0.021 (2)	0.0204 (19)	0.0145 (19)	0.0067 (16)	0.0039 (16)	0.0062 (16)
C8	0.026 (2)	0.032 (2)	0.019 (2)	0.0132 (18)	0.0099 (17)	0.0130 (18)
C9	0.021 (2)	0.033 (2)	0.020 (2)	0.0128 (18)	0.0040 (17)	0.0171 (18)
C10	0.025 (2)	0.023 (2)	0.0137 (19)	0.0021 (17)	0.0042 (17)	0.0087 (16)
C11	0.026 (2)	0.027 (2)	0.018 (2)	0.0089 (18)	0.0097 (17)	0.0090 (17)
C12	0.023 (2)	0.025 (2)	0.016 (2)	0.0109 (17)	0.0050 (16)	0.0077 (16)
C13	0.034 (3)	0.037 (2)	0.017 (2)	0.009 (2)	0.0023 (18)	0.0198 (19)
C14	0.020 (2)	0.0241 (19)	0.0157 (19)	0.0098 (16)	0.0079 (16)	0.0120 (16)
C15	0.029 (2)	0.026 (2)	0.0137 (19)	0.0125 (18)	0.0060 (17)	0.0097 (16)
C16	0.036 (3)	0.032 (2)	0.017 (2)	0.023 (2)	0.0105 (18)	0.0102 (18)
C17	0.036 (3)	0.042 (2)	0.022 (2)	0.026 (2)	0.0203 (19)	0.0186 (19)
C18	0.021 (2)	0.039 (2)	0.025 (2)	0.0130 (19)	0.0080 (18)	0.018 (2)
C19	0.024 (2)	0.028 (2)	0.0142 (19)	0.0108 (17)	0.0063 (16)	0.0094 (17)
C20	0.0149 (19)	0.027 (2)	0.0157 (19)	0.0104 (16)	0.0064 (15)	0.0104 (16)
C21	0.026 (2)	0.042 (3)	0.022 (2)	0.008 (2)	0.0134 (18)	0.016 (2)
C22	0.036 (3)	0.047 (3)	0.046 (3)	0.013 (2)	0.019 (2)	0.030 (2)
C23	0.021 (2)	0.025 (2)	0.0168 (19)	0.0100 (17)	0.0095 (16)	0.0090 (16)
C24	0.025 (2)	0.0185 (18)	0.022 (2)	0.0072 (16)	0.0106 (17)	0.0106 (16)
C25	0.032 (2)	0.021 (2)	0.019 (2)	0.0095 (18)	0.0115 (18)	0.0070 (17)
C26	0.028 (2)	0.022 (2)	0.016 (2)	0.0072 (18)	0.0065 (17)	0.0037 (17)
C27	0.032 (3)	0.029 (2)	0.020 (2)	0.0041 (19)	0.0109 (19)	0.0103 (18)
C28	0.042 (3)	0.027 (2)	0.014 (2)	0.006 (2)	0.0066 (19)	0.0063 (17)
C29	0.037 (3)	0.036 (2)	0.019 (2)	0.015 (2)	0.0140 (19)	0.0139 (19)
C30	0.036 (3)	0.026 (2)	0.023 (2)	0.0109 (19)	0.0135 (19)	0.0120 (18)
C31	0.032 (2)	0.025 (2)	0.018 (2)	0.0076 (18)	0.0114 (18)	0.0096 (17)
C32	0.073 (4)	0.047 (3)	0.015 (2)	0.004 (3)	0.014 (2)	0.008 (2)
C33	0.025 (2)	0.024 (2)	0.0129 (19)	0.0068 (17)	0.0072 (16)	0.0075 (16)
C34	0.030 (3)	0.032 (2)	0.037 (3)	0.012 (2)	0.016 (2)	0.017 (2)
C35	0.037 (3)	0.033 (2)	0.046 (3)	0.014 (2)	0.010 (2)	0.025 (2)
C36	0.036 (3)	0.027 (2)	0.027 (2)	0.0022 (19)	0.009 (2)	0.0163 (19)
C37	0.030 (3)	0.034 (2)	0.026 (2)	0.005 (2)	0.012 (2)	0.013 (2)
C38	0.031 (3)	0.026 (2)	0.028 (2)	0.0095 (18)	0.0146 (19)	0.0142 (18)

### Geometric parameters (Å, º)

**Table:**

Zn—N3	2.041 (3)	C14—C15	1.395 (5)
Zn—N6	2.071 (3)	C14—C19	1.397 (5)
Zn—S1	2.2879 (11)	C15—C16	1.382 (6)
Zn—S2	2.2757 (11)	C15—H15	0.9500
C1—N2	1.314 (5)	C16—C17	1.381 (6)
C4—N3	1.310 (5)	C16—H16	0.9500
C5—C6	1.349 (5)	C17—C18	1.383 (6)
C20—N5	1.307 (5)	C17—H17	0.9500
C23—N6	1.319 (5)	C18—C19	1.384 (6)
C24—C25	1.344 (5)	C18—H18	0.9500
S2—C20	1.768 (4)	C19—H19	0.9500
O1—C10	1.365 (5)	C21—C22	1.524 (6)
O1—C13	1.433 (5)	C21—H21A	0.9900
O2—C29	1.366 (5)	C21—H21B	0.9900
O2—C32	1.438 (5)	C22—H22A	0.9800
N1—C1	1.352 (5)	C22—H22B	0.9800
N1—C2	1.453 (6)	C22—H22C	0.9800
N1—H1N	0.85 (4)	C23—C24	1.441 (6)
N2—N3	1.385 (4)	C23—C33	1.499 (6)
N4—C20	1.361 (5)	C24—H24	0.9500
N4—C21	1.467 (6)	C25—C26	1.463 (6)
N4—H4N	0.83 (4)	C25—H25	0.9500
N5—N6	1.386 (5)	C26—C31	1.396 (6)
C2—C3	1.500 (7)	C26—C27	1.403 (5)
C2—H2A	0.9900	C27—C28	1.391 (6)
C2—H2B	0.9900	C27—H27	0.9500
C3—H3A	0.9800	C28—C29	1.382 (6)
C3—H3B	0.9800	C28—H28	0.9500
C3—H3C	0.9800	C29—C30	1.394 (6)
C4—C5	1.449 (5)	C30—C31	1.373 (6)
C4—C14	1.486 (5)	C30—H30	0.9500
C5—H5	0.9500	C31—H31	0.9500
C6—C7	1.465 (5)	C32—H32A	0.9800
C6—H6	0.9500	C32—H32B	0.9800
C7—C8	1.396 (6)	C32—H32C	0.9800
C7—C12	1.396 (6)	C33—C38	1.383 (6)
C8—C9	1.394 (6)	C33—C34	1.385 (6)
C8—H8	0.9500	C34—C35	1.394 (7)
C9—C10	1.382 (6)	C34—H34	0.9500
C9—H9	0.9500	C35—C36	1.368 (7)
C10—C11	1.404 (6)	C35—H35	0.9500
C11—C12	1.367 (6)	C36—C37	1.382 (6)
C11—H11	0.9500	C36—H36	0.9500
C12—H12	0.9500	C37—C38	1.388 (6)
C13—H13A	0.9800	C37—H37	0.9500
C13—H13B	0.9800	C38—H38	0.9500
C13—H13C	0.9800		
			
N3—Zn—N6	107.16 (12)	C15—C16—C17	120.6 (4)
N3—Zn—S2	127.83 (9)	C15—C16—H16	119.7
N6—Zn—S2	86.73 (9)	C17—C16—H16	119.7
N3—Zn—S1	87.29 (9)	C16—C17—C18	119.8 (4)
N6—Zn—S1	121.90 (9)	C16—C17—H17	120.1
S2—Zn—S1	127.92 (4)	C18—C17—H17	120.1
C1—S1—Zn	92.45 (13)	C17—C18—C19	120.2 (4)
C20—S2—Zn	92.86 (13)	C17—C18—H18	119.9
C10—O1—C13	117.1 (3)	C19—C18—H18	119.9
C29—O2—C32	117.1 (4)	C18—C19—C14	120.3 (4)
C1—N1—C2	121.0 (3)	C18—C19—H19	119.9
C1—N1—H1N	118 (3)	C14—C19—H19	119.9
C2—N1—H1N	115 (3)	N5—C20—N4	116.9 (4)
C1—N2—N3	115.8 (3)	N5—C20—S2	128.0 (3)
C4—N3—N2	115.4 (3)	N4—C20—S2	115.1 (3)
C4—N3—Zn	127.7 (3)	N4—C21—C22	113.3 (4)
N2—N3—Zn	116.7 (2)	N4—C21—H21A	108.9
C20—N4—C21	121.3 (3)	C22—C21—H21A	108.9
C20—N4—H4N	111 (3)	N4—C21—H21B	108.9
C21—N4—H4N	120 (3)	C22—C21—H21B	108.9
C20—N5—N6	115.2 (3)	H21A—C21—H21B	107.7
C23—N6—N5	114.8 (3)	C21—C22—H22A	109.5
C23—N6—Zn	128.6 (3)	C21—C22—H22B	109.5
N5—N6—Zn	116.6 (2)	H22A—C22—H22B	109.5
N2—C1—N1	115.8 (4)	C21—C22—H22C	109.5
N2—C1—S1	127.4 (3)	H22A—C22—H22C	109.5
N1—C1—S1	116.8 (3)	H22B—C22—H22C	109.5
N1—C2—C3	111.2 (4)	N6—C23—C24	117.3 (4)
N1—C2—H2A	109.4	N6—C23—C33	120.4 (3)
C3—C2—H2A	109.4	C24—C23—C33	122.3 (3)
N1—C2—H2B	109.4	C25—C24—C23	125.5 (4)
C3—C2—H2B	109.4	C25—C24—H24	117.3
H2A—C2—H2B	108.0	C23—C24—H24	117.3
C2—C3—H3A	109.5	C24—C25—C26	124.5 (4)
C2—C3—H3B	109.5	C24—C25—H25	117.7
H3A—C3—H3B	109.5	C26—C25—H25	117.7
C2—C3—H3C	109.5	C31—C26—C27	116.6 (4)
H3A—C3—H3C	109.5	C31—C26—C25	123.6 (3)
H3B—C3—H3C	109.5	C27—C26—C25	119.8 (4)
N3—C4—C5	116.2 (3)	C28—C27—C26	121.6 (4)
N3—C4—C14	122.3 (3)	C28—C27—H27	119.2
C5—C4—C14	121.4 (3)	C26—C27—H27	119.2
C6—C5—C4	125.7 (4)	C29—C28—C27	119.9 (4)
C6—C5—H5	117.2	C29—C28—H28	120.1
C4—C5—H5	117.2	C27—C28—H28	120.1
C5—C6—C7	125.3 (4)	O2—C29—C28	125.4 (4)
C5—C6—H6	117.4	O2—C29—C30	114.9 (4)
C7—C6—H6	117.4	C28—C29—C30	119.7 (4)
C8—C7—C12	117.8 (4)	C31—C30—C29	119.6 (4)
C8—C7—C6	119.3 (4)	C31—C30—H30	120.2
C12—C7—C6	122.9 (4)	C29—C30—H30	120.2
C7—C8—C9	121.7 (4)	C30—C31—C26	122.7 (4)
C7—C8—H8	119.1	C30—C31—H31	118.7
C9—C8—H8	119.1	C26—C31—H31	118.7
C10—C9—C8	119.2 (4)	O2—C32—H32A	109.5
C10—C9—H9	120.4	O2—C32—H32B	109.5
C8—C9—H9	120.4	H32A—C32—H32B	109.5
O1—C10—C9	124.7 (4)	O2—C32—H32C	109.5
O1—C10—C11	115.8 (4)	H32A—C32—H32C	109.5
C9—C10—C11	119.5 (4)	H32B—C32—H32C	109.5
C12—C11—C10	120.6 (4)	C38—C33—C34	119.4 (4)
C12—C11—H11	119.7	C38—C33—C23	120.9 (4)
C10—C11—H11	119.7	C34—C33—C23	119.7 (4)
C11—C12—C7	121.1 (4)	C33—C34—C35	119.6 (5)
C11—C12—H12	119.4	C33—C34—H34	120.2
C7—C12—H12	119.4	C35—C34—H34	120.2
O1—C13—H13A	109.5	C36—C35—C34	121.0 (4)
O1—C13—H13B	109.5	C36—C35—H35	119.5
H13A—C13—H13B	109.5	C34—C35—H35	119.5
O1—C13—H13C	109.5	C35—C36—C37	119.5 (4)
H13A—C13—H13C	109.5	C35—C36—H36	120.2
H13B—C13—H13C	109.5	C37—C36—H36	120.2
C15—C14—C19	119.1 (4)	C38—C37—C36	120.0 (4)
C15—C14—C4	120.4 (3)	C38—C37—H37	120.0
C19—C14—C4	120.5 (3)	C36—C37—H37	120.0
C16—C15—C14	120.0 (4)	C33—C38—C37	120.5 (4)
C16—C15—H15	120.0	C33—C38—H38	119.7
C14—C15—H15	120.0	C37—C38—H38	119.7
			
C1—N2—N3—C4	−178.6 (3)	C15—C14—C19—C18	0.3 (6)
C1—N2—N3—Zn	6.6 (4)	C4—C14—C19—C18	179.8 (4)
C20—N5—N6—C23	−171.6 (3)	N6—N5—C20—N4	−179.2 (3)
C20—N5—N6—Zn	6.6 (4)	N6—N5—C20—S2	−0.3 (5)
N3—N2—C1—N1	179.8 (3)	C21—N4—C20—N5	−7.9 (5)
N3—N2—C1—S1	−2.8 (5)	C21—N4—C20—S2	173.0 (3)
C2—N1—C1—N2	−9.3 (5)	Zn—S2—C20—N5	−4.9 (4)
C2—N1—C1—S1	173.0 (3)	Zn—S2—C20—N4	174.0 (3)
Zn—S1—C1—N2	−1.7 (3)	C20—N4—C21—C22	80.6 (5)
Zn—S1—C1—N1	175.7 (3)	N5—N6—C23—C24	178.6 (3)
C1—N1—C2—C3	−180.0 (4)	Zn—N6—C23—C24	0.7 (5)
N2—N3—C4—C5	174.4 (3)	N5—N6—C23—C33	0.8 (5)
Zn—N3—C4—C5	−11.5 (5)	Zn—N6—C23—C33	−177.1 (3)
N2—N3—C4—C14	−3.8 (5)	N6—C23—C24—C25	−168.2 (4)
Zn—N3—C4—C14	170.3 (2)	C33—C23—C24—C25	9.6 (6)
N3—C4—C5—C6	176.5 (4)	C23—C24—C25—C26	173.3 (4)
C14—C4—C5—C6	−5.2 (6)	C24—C25—C26—C31	−4.0 (7)
C4—C5—C6—C7	177.4 (4)	C24—C25—C26—C27	178.6 (4)
C5—C6—C7—C8	179.1 (4)	C31—C26—C27—C28	−0.4 (7)
C5—C6—C7—C12	−2.0 (6)	C25—C26—C27—C28	177.2 (4)
C12—C7—C8—C9	0.7 (6)	C26—C27—C28—C29	−0.2 (7)
C6—C7—C8—C9	179.6 (4)	C32—O2—C29—C28	−6.2 (7)
C7—C8—C9—C10	−0.9 (6)	C32—O2—C29—C30	174.5 (4)
C13—O1—C10—C9	−0.9 (6)	C27—C28—C29—O2	−179.1 (4)
C13—O1—C10—C11	179.5 (3)	C27—C28—C29—C30	0.2 (7)
C8—C9—C10—O1	−179.5 (4)	O2—C29—C30—C31	179.7 (4)
C8—C9—C10—C11	0.1 (6)	C28—C29—C30—C31	0.3 (7)
O1—C10—C11—C12	−179.6 (4)	C29—C30—C31—C26	−0.9 (7)
C9—C10—C11—C12	0.8 (6)	C27—C26—C31—C30	1.0 (7)
C10—C11—C12—C7	−1.0 (6)	C25—C26—C31—C30	−176.5 (4)
C8—C7—C12—C11	0.3 (6)	N6—C23—C33—C38	70.3 (5)
C6—C7—C12—C11	−178.6 (4)	C24—C23—C33—C38	−107.4 (5)
N3—C4—C14—C15	−55.3 (5)	N6—C23—C33—C34	−107.7 (4)
C5—C4—C14—C15	126.6 (4)	C24—C23—C33—C34	74.6 (5)
N3—C4—C14—C19	125.3 (4)	C38—C33—C34—C35	−1.7 (6)
C5—C4—C14—C19	−52.9 (5)	C23—C33—C34—C35	176.4 (4)
C19—C14—C15—C16	0.3 (6)	C33—C34—C35—C36	1.0 (7)
C4—C14—C15—C16	−179.1 (4)	C34—C35—C36—C37	0.4 (7)
C14—C15—C16—C17	−1.3 (6)	C35—C36—C37—C38	−1.0 (7)
C15—C16—C17—C18	1.7 (7)	C34—C33—C38—C37	1.1 (6)
C16—C17—C18—C19	−1.1 (7)	C23—C33—C38—C37	−176.9 (4)
C17—C18—C19—C14	0.1 (6)	C36—C37—C38—C33	0.2 (6)

### Hydrogen-bond geometry (Å, º)

Cg1—Cg4 are the centroids of the (C33–C38), (Zn,S2,C20,N5,N6), (C26—C31) and (Zn,S1,C1,N2,N3) rings, respectively.

**Table:**

*D*—H···*A*	*D*—H	H···*A*	*D*···*A*	*D*—H···*A*
N1—H1*N*···S1^i^	0.85 (5)	2.66 (5)	3.506 (4)	171 (3)
N4—H4*N*···S2^ii^	0.84 (5)	2.82 (5)	3.477 (5)	137 (4)
C36—H36···O1^iii^	0.95	2.57	3.428 (6)	151
C16—H16···*Cg*1^iv^	0.95	2.85	3.747 (4)	157
C18—H18···*Cg*2^v^	0.95	2.69	3.485 (5)	141
C34—H34···*Cg*3^iv^	0.95	2.72	3.555 (6)	148
C5—H5···*Cg*2	0.95	2.67	3.462 (5)	142
C24—H24···*Cg*4	0.95	2.55	3.421 (5)	153

Symmetry codes: (i) −*x*+1, −*y*+1, −*z*; (ii) −*x*+1, −*y*+1, −*z*+1; (iii) −*x*+1, −*y*, −*z*+1; (iv) −*x*+1, −*y*, −*z*; (v) *x*+1, *y*, *z*.

## References

[bb1] Afrasiabi, Z., Sinn, E., Padhye, S., Dutta, S., Padhye, S., Newton, C., Anson, C. E. & Powell, A. K. (2003). *J. Inorg. Biochem* **95**, 306–314. 10.1016/s0162-0134(03)00131-4 12818801

[bb2] Agilent (2013). *CrysAlis PRO*. Agilent Technologies, Yarnton, England.

[bb3] Bisceglie, F., Tavone, M., Mussi, F., Azzoni, S., Montalbano, S., Franzoni, S., Tarasconi, P., Buschini, A. & Pelosi, G. (2018). *J. Inorg. Biochem.* **179**, 60–70. 10.1016/j.jinorgbio.2017.11.009 29175629

[bb4] Brandenburg, K. (2006). *DIAMOND*. Crystal Impact GbR, Bonn, Germany.

[bb5] Dilworth, J. R. & Hueting, R. (2012). *Inorg. Chim. Acta*, **389**, 3–15.

[bb6] Espíndola, J. W. P., Cardoso, M. V. de O., Filho, G. B. de O., Silva, D. A. O e, Moreira, D. R. M., Bastos, T. M., de Simone, C. A., Soares, M. B. P., Villela, F. S., Ferreira, R. S., de Castro, M. C. A. B., Pereira, V. R. A., Murta, S. M. F., Sales Junior, P. A., Romanha, A. J. & Leite, A. C. L. (2015). *Eur. J. Med. Chem.* **101**, 818–835. 10.1016/j.ejmech.2015.06.048 26231082

[bb7] Farrugia, L. J. (2012). *J. Appl. Cryst.* **45**, 849–854.

[bb8] Garoufis, A., Hadjikakou, S. K. & Hadjiliadis, N. (2009). *Coord. Chem. Rev.* **253**, 1384–1397.

[bb9] Lobana, T. S., Sharma, R., Bawa, G. & Khanna, S. (2009). *Coord. Chem. Rev.* **253**, 977–1055.

[bb10] Low, M. L., Maigre, L. M., Tahir, M. I. M. T., Tiekink, E. R. T., Dorlet, P., Guillot, R., Ravoof, T. B., Rosli, R., Pagès, J.-M., Policar, C., Delsuc, N. & Crouse, K. A. (2016). *Eur. J. Med. Chem.* **120**, 1–12. 10.1016/j.ejmech.2016.04.027 27183379

[bb11] Malenov, D. P., Janjić, G. V., Medaković, V. B., Hall, M. B. & Zarić, S. D. (2017). *Coord. Chem. Rev.* **345**, 318–341.

[bb12] McKinnon, J. J., Jayatilaka, D. & Spackman, M. A. (2007). *Chem. Commun.* pp. 3814–3816. 10.1039/b704980c 18217656

[bb14] Palusiak, M. & Krygowski, T. M. (2007). *Chem. Eur. J.* **13**, 7996–8006. 10.1002/chem.200700250 17607686

[bb15] Pelivan, K., Miklos, W., van Schoonhoven, S., Koellensperger, G., Gille, L., Berger, W., Heffeter, P., Kowol, C. R. & Keppler, B. K. (2016). *J. Inorg. Biochem.* **160**, 61–69. 10.1016/j.jinorgbio.2015.10.006 26507768

[bb16] Quiroga, A. G. & Ranninger, C. N. (2004). *Coord. Chem. Rev* **248**, 119–133.

[bb17] Sheldrick, G. M. (2008). *Acta Cryst.* A**64**, 112–122. 10.1107/S0108767307043930 18156677

[bb18] Sheldrick, G. M. (2015). *Acta Cryst.* C**71**, 3–8.

[bb19] Tan, M. Y., Crouse, K. A., Ravoof, T. B. S. A., Jotani, M. M. & Tiekink, E. R. T. (2017). *Acta Cryst.* E**73**, 1001–1008. 10.1107/S2056989017008064 PMC5499278 28775870

[bb20] Tiekink, E. R. T. (2017). *Coord. Chem. Rev.* **345**, 209–228.

[bb21] Westrip, S. P. (2010). *J. Appl. Cryst.* **43**, 920–925.

[bb22] Yang, L., Powell, D. R. & Houser, R. P. (2007). *Dalton Trans.* pp. 955–964. 10.1039/b617136b 17308676

[bb23] Yeo, C. I., Halim, S. N. A., Ng, S. W., Tan, S. L., Zukerman-Schpector, J., Ferreira, M. A. B. & Tiekink, E. R. T. (2014). *Chem. Commun.* **50**, 5984–5986. 10.1039/c4cc02040e 24763907

[bb13] Yusof, E. N. M., Ravoof, T. B. S. A., Tiekink, E. R. T., Veerakumarasivam, A., Crouse, K. A., Tahir, M. I. M. & Ahmad, H. (2015). *Int. J. Mol. Sci* **16**, 11034–11054. 10.3390/ijms160511034 PMC4463689 25988384

[bb24] Zukerman-Schpector, J., Yeo, C. I. & Tiekink, E. R. T. (2016). *Z. Kristallogr* **231**, 55–64.

